# An Automatic Car Counting System Using OverFeat Framework

**DOI:** 10.3390/s17071535

**Published:** 2017-06-30

**Authors:** Debojit Biswas, Hongbo Su, Chengyi Wang, Jason Blankenship, Aleksandar Stevanovic

**Affiliations:** 1 Department of Civil, Environmental and Geomatics Engineering, Florida Atlantic University, 777 Glades Rd, Boca Raton, FL 33431, USA; dbiswas2015@fau.edu (D.B.); jblankenship2014@fau.edu (J.B.); astevano@fau.edu (A.S.); 2 Institute of Remote Sensing and Digital Earth, Chinese Academy of Sciences, No. 9 Dengzhuang South Road, Haidian District, Beijing 100094, China; wangcycastle@163.com

**Keywords:** car counting, OverFeat Framework, Background Subtraction Method, Placemeter, Convolution Neural Network

## Abstract

Automatic car counting is an important component in the automated traffic system. Car counting is very important to understand the traffic load and optimize the traffic signals. In this paper, we implemented the Gaussian Background Subtraction Method and OverFeat Framework to count cars. OverFeat Framework is a combination of Convolution Neural Network (CNN) and one machine learning classifier (like Support Vector Machines (SVM) or Logistic Regression). With this study, we showed another possible application area for the OverFeat Framework. The advantages and shortcomings of the Background Subtraction Method and OverFeat Framework were analyzed using six individual traffic videos with different perspectives, such as camera angles, weather conditions and time of the day. In addition, we compared the two algorithms above with manual counting and a commercial software called Placemeter. The OverFeat Framework showed significant potential in the field of car counting with the average accuracy of 96.55% in our experiment.

## 1. Introduction

In today’s world, properly maintaining the traffic system is a very tedious job. Every day, the number of vehicles increases at exponential order [1]. We need to make our road transportation system automated as much as possible so that traffic can move smoothly without much manual intervention. Automatic car counting is one of the fundamental tasks for intelligent traffic surveillance system. Traffic flow is required in the urban transportation system because its estimation is helpful in evaluating traffic state for management. Automatic vehicle counting is a key technique to monitor and estimate traffic flow. Therefore, car counting is important and helpful to optimize the traffic signaling system [2,3]. In addition, it helps to redirect the traffic to alternate less congested roads on a demanding day with special events. Understanding the physical traffic load is an important application of car counting. This enables an opportunity for transportation engineers and decision makers to plan their budget well before renovating an existing road or building a new road depending on the car density statistics. Counting could be done by radar, infrared or inductive loop detectors, besides the use of traffic cameras. Although some technologies have shown a viable performance to count cars, they could also be intrusive and have a higher maintenance cost. A computer vision based system could be a suitable alternative for car counting. However, current vision-based systems are limited to weather condition and natural lights [3].

Our purpose in this study is to develop an automated car counting system from traffic videos that can perform well in both day and night, in sunny and cloudy weather conditions. We took into account the vibration effect caused by cameras being installed on a bridge or similar conditions. Background Subtraction Method (BSM) and OverFeat Framework [4] have been implemented in our study. The performance measure of BSM and OverFeat Framework has been evaluated with manual counting and Placemeter. The OverFeat framework has shown the best performance in terms of car detection (Avg. accuracy 96.55%) for videos of poor resolution and taken in adverse daylight conditions.

## 2. Related Work

A brief survey of the related work in intelligent traffic monitoring system using traffic cameras is presented in the section of Buch et al. [3]. Daigavane and Bajaj [5] presented a background registration technique and segmentation using a morphological operator. In this study, a system has been developed to detect and count objects dynamically on highways. The system effectively combines simple domain knowledge about object classes with time domain statistical measures to identify target objects in the presence of partial occlusions and ambiguous poses. Chen et al. [6] addressed the issues regarding unsupervised image segmentation and object modeling with multimedia inputs to capture the spatial and temporal behavior of the object for traffic monitoring. Gupte et al. [7] showed algorithms for vision-based detection and classification of vehicles in monocular image sequences of traffic scenes that are recorded by a stationary camera. Processing is done at three levels: raw images, region level and vehicle level. Vehicles are modelled as rectangular patterns with certain dynamic behavior. Cheung and Kamath [8] compared the performance of a large set of different background models on urban traffic videos. They experimented with sequences filmed in weather conditions such as snow and fog, for which a robust background model is required. Kanhere et al. [9] applied a feature tracking approach to traffic viewed from a low-angle off axis camera. Vehicle occlusions and perspective effects pose a more significant challenge for a camera placed low to the ground. Deva et al. [10] proposed a concept to automatically track the articulations of people from video sequences. This is a challenging task but contains a rich body of relevant literature. It can identify and track individuals and count distinct people. Toufiq et al. [11] described background subtraction as the widely-used paradigm for detection of moving objects in videos taken from a static camera that has a very wide range of applications. The main idea behind this concept is to automatically generate and maintain a representation of the background, which can be later used to classify any new observation as background or foreground. 

Gao et al. [12] research showed that a set of Scale Invariant Feature Transform (SIFT) features are extracted and matched in the follow-up image frames to improve tracking performance for more accurate detection. The SIFT features are also detected, tracked and clustered in the foreground blobs in Jun et al. [13]. Horizontal and vertical line features are extracted to build a 3D vehicle model at Leotta and Mundy’s [14] work, assuming the vehicle is not occluded, by predicting and matching image intensity edges to fit a generic 3D vehicle model to multiple still images. Simultaneous tracking can also be done during the shape estimation in a video. Ma and Grimson [15] proposed a vehicle classification algorithm that uses the feature based on edge points and modified SIFT descriptors. Two classification tasks, cars versus minivans and sedans versus taxies, are tested with satisfactory performance. Buch et al. proposed a 3D extended Histograms of Oriented Gradients (HOG) feature for detection and classification of individual vehicles and pedestrians by combining 3D interest points and HOG. 3D vehicle models are pre-reconstructed by the methods in Messelodi et al. [16]. Hsieh et al. [2] further extracted region size and vehicle “linearity” to classify vehicles into four categories (e.g., car, minivan, truck and van truck), assuming that individual vehicles have been separated after lane and shadow detection. Alonso et al. [17] extracted image regions according to high edge density areas. Shadows, symmetry measurement and Harris corners are then used in the hypothesis classification. Lou et al. [18] extracted image regions of interest based on motion detection. Then, a 3D model is fitted to the image region using a point-to-line segment distance metric. Occlusion is a major challenging problem in the vehicle segmentation. Many methods have been proposed to deal with this problem. Features mentioned above could be considered as a set of “parts” that are tracked and grouped together [9,18]. When the 2D/3D vehicle model could be fitted into image frames, it is also relatively easy to detect occlusions [19,20]. Liang et al. [21] extracted a “cutting region” between two occluded vehicles based on the motion field of consecutive image frames. Similarly, a “cutting line” is estimated in a Traffic Scorecard [1] to separate two occluded vehicles based on the analysis of convex shape. Image warping is not considered as a step or module to detect and track vehicles in Buch et al. [3]. There is some research using image warping as a pre-processing step to generate a horizontal or vertical road segment to facilitate the detection and tracking (e.g., [13,22]). Four reference points are selected to estimate a projective transformation in Jun et al. [13]. This transformation is applied so that all motion vectors are approximately parallel to each other. The similar idea is applied in Salvi et al. [22] so that lanes could be detected easily. However, image warping itself has not been applied directly to detect unclassified vehicles. Liang et al. [21] applied a cascaded regression model to count and classify vehicles directly. They have shown the algorithm can deal with the traffic with severe occlusions and very low vehicle resolutions.

This paper is organized as follows. First, the implementation of BSM and OverFeat Framework is discussed in Section 3. Section 3 also described the process of manual counting and information about Placemeter. Section 4 contains the information about the dataset used. The experimental results, where an analysis between the BSM, OverFeat Framework, Placemeter and manual counting performed to verify the quality of our implemented method, are presented in Section 5. Finally, Section 6 is devoted to the conclusions and discussion of future work.

## 3. Methodology

### 3.1. Background Subtraction Method

**Figure 1:**
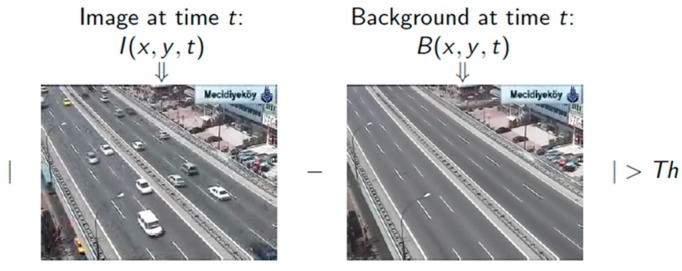
Basic approach of background subtraction method.

Background Subtraction Method is a simplistic but effective method of finding moving objects from a frame. The basic concept of the algorithm is finding frame difference and applying a threshold to get the moving object of interest. In Figure 1, B (*x*, *y*, *t*) denotes a background frame at time *t* and I (*x*, *y*, *t*) denotes target frame at time *t*. After the frame differences are done, moving objects are extracted depending on the threshold value.

Mean filter and median filter are two other approaches of the background subtraction method. Among the first *n* number of frames, mean background frame is calculated for the mean filter and median background frame is calculated for the median filter. The advantages of these algorithms are that they are very easy to implement and use. Furthermore, the background is dynamic. Nevertheless, the accuracy of frame differencing depends on the object speed and frame rate. Mean and median background models have relatively high memory requirements. Another major problem with these approaches is that there is only one threshold and the threshold is not a function of time. These models also do not work for the bimodal system. However, the Gaussian Mixture Model (GMM) can overcome these issues.

#### 3.1.1. Gaussian Mixture Model (GMM)

We have implemented the GMM for our study after experiencing the drawbacks of the existing background subtraction method. The steps to implement the GMM are as below [23]:

- model the values of a particular pixel as a mixture of Gaussians;
- determine which Gaussians may correspond to background colors-based on the persistence and the variance of each of the Gaussians;
- pixel values that do not fit the background distributions are considered foreground until there is a Gaussian that includes them;
- update the Gaussians;
- pixel values that do not match one of the pixel’s “background” Gaussians are grouped using connected components.

Modeling pixel values can be done using the steps listed below. At time *t*, if there is *k* distribution of Gaussian for each pixel, the value of *k* is determined by using information-theoretic criteria (Bayesian information criterion (BIC)). BIC is a benchmark for model selection among a finite set of models; the model with the lowest BIC is preferred. BIC is based on likelihood function.

Each Gaussian contains:

- $\omega_{i,t}$ is an estimate of the weight of *i*th Gaussian in the mixture at time *t* (the portion of data accounted for by this Gaussian). Initially, we considered that all the Gaussians have the same weights.
- ${µ}_{i,t}$ is the mean value of the *i*th Gaussian in the mixture at time *t*.
- $\sum_{i,t}$ is the covariance matrix of the *i*th Gaussian in the mixture at time *t*.

The Gaussian probability density function is η ($X_{t}$, µ, ∑). The probability of observing the current pixel value is:

(1) $$P\left(X_{t}\right)=\sum_{i=1}^{k}\omega_{i,t}\;\times \;\eta (X_{t}{,\;µ}_{i,t},\;\sum_{i,t})\;.$$

There are four stages to update the mixture model.

**Stage 1:**

- Every new pixel value, $X_{t}$ is checked against the existing *k* Gaussian distributions until a match is found.

A match is defined as a pixel value within 2.5 standard deviations of a distribution. Within the 2.5 standard deviation, almost 99.4% of data is used to define the pixels. 

**Stage 2—No match:**

- If none of the *k* distributions match the current pixel value, the least probable distribution is discarded.
- A new distribution with the current value as its mean value, and an initially high variance and low prior weight is entered.

**Stage 3:**

- The prior weights of the *k* distribution at time *t* are adjusted as follows:
  (2) $$\omega_{k,t}=\left(1-\alpha \right)\omega_{k,t-1}+\alpha \left(M_{k,t}\right),$$

where α is the learning rate and $M_{k,t}$ is 1 for the model where matched and 0 for the remaining models. 

**Stage 4:**

The µ and σ parameters for unmatched distributions remain same;

The parameters of the distribution that match the new observation are updated as follows:

(3) $${µ}_{t}=\left(1-\rho \right){µ}_{t-1}+\rho \left(X_{t}\right),$$

(4) $$\sigma_{t}^{2}=\left(1-\rho \right)\sigma_{t-1}^{2}+\rho {\left(X_{t}-{µ}_{t}\right)}^{T}\left(X_{t}-{µ}_{t}\right),$$

where $\sigma_{t-1}^{2}$ is the last variance and $\rho {\left(X_{t}-{µ}_{t}\right)}^{T}\left(X_{t}-{µ}_{t}\right)$ is the distance of the new pixel from the updated mean.

#### 3.1.2. Implementation of the Background Subtraction Method

BSM was applied to the experiment video frames with RGB color space. Then, morphological operation was performed. An opening operation has been performed to remove small blobs from the frames. The bright spots are the region of interest, so a threshold value has been applied to discover bright pixels. Then, a find contour operation is done on binary frames, which returns the blocks of bright pixels. For example, a binary frame might have twenty blocks of bright/change pixels including small, medium and large blocks. We calculated the contour area applying a max-area and min-area threshold depending on the car size to identify the car contours. The find contour operation then returns five contours, which indicate cars only. 

After the contours are computed, the centroid of each car is calculated. A count line has been drawn to count the cars. When the centroid of the car crosses the count line, the car count increases by one. The algorithm was implemented using Python 2.7 with an OpenCV 2.4.10 library because of its cross-platform compatibility and strong image processing library. 

### 3.2. OverFeat Framework

OverFeat Framework is a combination of Convolution Neural Network (CNN) and another machine learning classifier (logistic regression (LR), support vector machines (SVM), etc.). The training part of CNN is cumbersome [4]. It is slow and needs a large amount of training data. This is why CNN has been used for feature extraction only. The extracted feature information was provided to another machine learning classifier, which does not need a substantial amount of training data. We have used LR for classification in our study. In the next section, the detailed implementation of OverFeat Framework will be discussed.

#### 3.2.1. Convolution Neural Network (CNN) 

CNN is very effective in the field of image classification and recognition [24]. This special type of Neural Network was limited due to hardware capacity before 2010, as CNN demands a large amount of memory to process the huge amount of training data. Now, we have a solid-state hard drive with high-end graphic cards and an extended amount of memory, which gives an extra edge to run CNN algorithms efficiently. 

**Figure 2:**
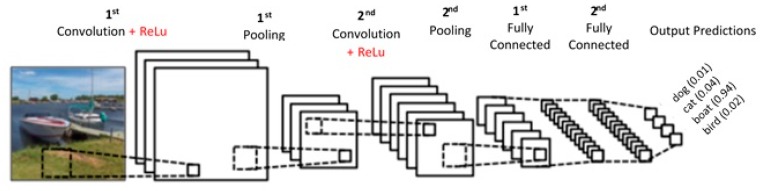
A simple CNN architecture.

CNN has several layers of convolution with nonlinear activation functions; like ReLu, tanh, sigmoid, etc as described in Figure 2. Unlike a traditional Neural Network, which is fully connected, CNN is regionally connected. A region of input is connected to a region of output. Different sets of filters are applied to each layer of CNN to extract features. As we go deep into the layers, the number of filters increase to extract more detail features. From raw pixels, the edges are detected at the first level. Then, the shapes are detected from the edges and further detail information is detected that is specific to a particular object, like the difference between trucks and vans.

There are four main operations in the CNN:

- Convolution
- Nonlinearity (ReLu)
- Pooling
- Fully-connected layer (FC)

##### Convolution

**Figure 3:**
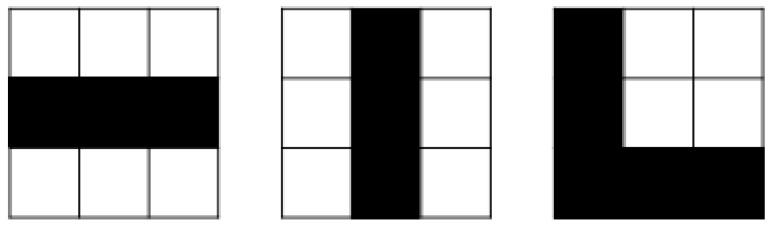
The filter on the left might activate strongest when it encounters a horizontal line; the one in the middle for a vertical line and the right one for ‘L’ shape line.

The convolution layer is the building block of CNN. Feature extraction is the main purpose of this layer. This layer consists of distinct types of filters like the vertical filter, horizontal filter, ‘X’ shape filter, ‘L’ shape filter, etc as described in Figure 3. 

**Figure 4:**
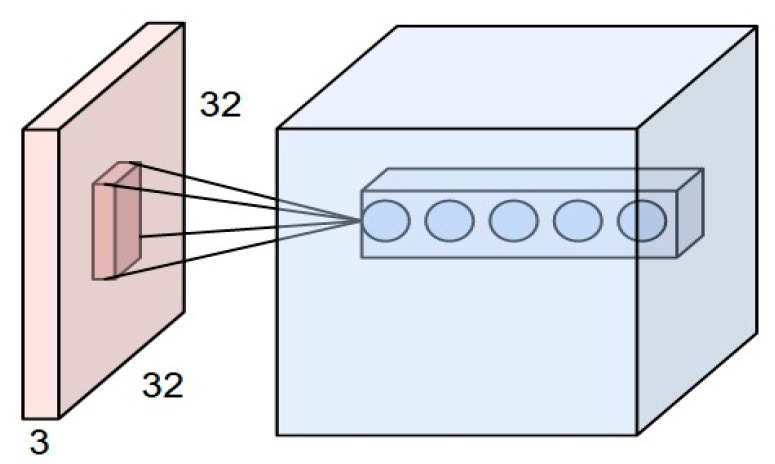
Convolving a small region of an image with a set of 5 filters of size F × F.

As we have discussed, the filters are responsible for extracting the features. We use different types of filters in the same region to extract the exact shapes. Figure 4 describes the concept more clearly. Let us assume, convolving a small region of the image, which the filter size is 3 × 3, and, as there are five filters, the dimension of the matrix after the operation will be 3 × 3 × 5.

##### Nonlinearity (ReLu)

The next operation is to apply a nonlinear function for learning. There are lots of nonlinear functions already available like sigmoid, tanh, etc. The rectified linear unit (ReLu) is used widely among the neural network community because it has solved the vanishing gradient problem. ReLu is a simple max function *f*(*x*) = max (0, *x*) applied after every convolution operation.

##### Pooling

**Figure 5:**
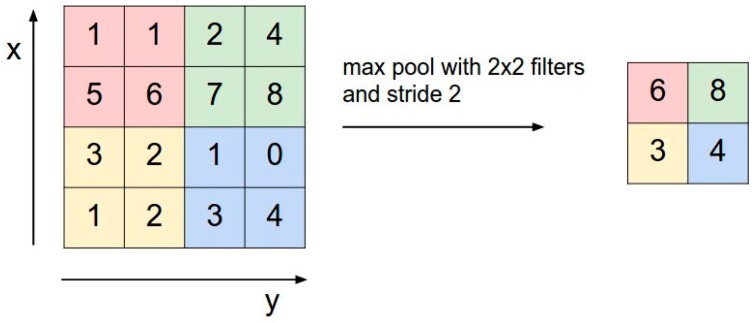
Max pooling over a 2 × 2 region with stride of 2.

The next layer is the pooling layer. The pooling reduces the spatial size of the input volume, which helps to reduce the amount of parameters and computation in the network. Pooling helps to control the overfitting problem. Figure 5 describes a max pooling with a 2 × 2 filter and stride 2.

##### Fully-Connected Layer (FC)

**Figure 6:**
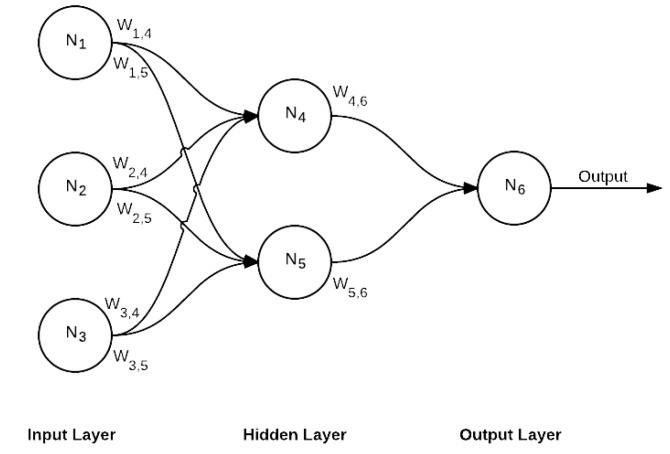
Fully connected layer.

FC is the final layer of this network, which is also a decision layer. This is the standard network where every neuron of the previous layer is connected to every neuron in the next layer, as shown in Figure 6.

The network may have multiple convolution layers, pooling layers and fully connected layers. The most common CNN architecture pattern is as below:

INPUT => [[CONV => RELU] × N => POOL?] × M => [FC => RELU] × K => FC,

where 0 ≤ N ≤ 3, M ≥ 0 and 0 ≤ K ≤ 3

The architecture that has been used for this study is discussed below.

#### 3.2.2. OverFeat Architecture

The OverFeat architecture used for the study is shown below.

Table 1 shows the Architecture of the OverFeat network. The framework has eight layers. The first layer consists of 96 filters of size 11 × 11 and the pool size is 2 × 2. ReLu has been applied after each convolution layer, which is not shown in Table 1. The second layer consists of 256 filters with size 5 × 5 and so on. We can notice that, as we go deep into the network, the number of filters increases and filter size decreases. This is because the deep layers extract more detailed information. The final layer is the fully connected layer. The architecture we have used is suggested by Zhang et al. [4] in their paper. We used FC 1 only (i.e., up to layer 6) for our study, as we want to reduce the computation. In addition, as soon as our desired accuracy (more than 95%) is achieved, we stop to minimize the computation.

#### 3.2.3. Implementation of the OverFeat Framework

There are three steps for the OverFeat framework setup: feature extraction, training and testing. The Convolution Neural Network has been used for feature extraction. The HDF5 data format has been used to save the extracted features. Six different types of cars and road data have been used for training. We have used up to six layers of deep level from Table 1, which shows the architecture of OverFeat network. The feature extracted database is now ready to use for training purposes. Any machine learning classifier could be used for the training. We have used logistic regression for our study. There were 3072 different variables used in the logistic regression. In the logistic regression method, we train weights (also known as hyper-parameters) to classify the cars. Logistic Regression is being implemented via the LogisticRegression function by the scikit-learn library [25]. After the hyper-parameters are trained properly, the testing or evaluation step is performed. This is the last step where a live traffic camera can be used for car counting. We have selected a small portion of each lane as a Region of Interest (ROI). The OverFeat Framework checks the ROIs for cars at every video frame. If the car is found at the ROI, the count increases by one. Every ROI can detect one car at a time. This is why we have tried to make the ROIs small enough to only fit a single car.

### 3.3. Commercial Software (Placemeter)

We used a commercial software named Placemeter to assess the performance of our approach. Placemeter [26] was started in 2012 and acquired by Netgear (San Jose, CA, USA.) in December 2016. In order to use Placemeter, we had to upload our video data to their web portal. Placemeter then uses an in-house algorithm to count the vehicles. Much like we did, a count line or threshold is defined in order to count the vehicles. This, in turn, generates a count file for the particular video. The user does not have access to set initial settings or make any changes during the experiment.

### 3.4. Manual Counting

We have hired a student to count the cars manually for our project. The student has counted the cars on a regular interval basis. For high traffic videos like camera 73, he has taken the one-minute interval and for the lower traffic videos like camera 103, he has taken five-minute intervals. We have calculated the accuracy of Placemeter, BSM and OverFeat Framework by using the manual counting as a ground truth.

## 4. Datasets

This section contains the detailed description of the training and testing dataset. Training data is very important for any neural network approach to get desired results.

### 4.1. Training Data 

We used seven types of classes in this study for training purposes: six different car classes and one road class (3698 images). The car classes are bus (1300 images), sports car (1300 image), taxi (1300 images), truck (1568 images), fire rescue (1300 images) and frame car (398 images). Except for the frame car, the rest of the training images are collected from Stanford Image-net library [27]. The frame car images are collected from the video frames, used in this study, with visual interpretation. We have collected three thousand and fifteen (3015) car images form the data library and we have created one hundred and ninety-seven (197) images from the video frames. During the implementation of the algorithm, all six of the car classes are merged into a single class called “CAR”. As our target was to detect the cars only without further classifying with sub classes, we kept two main classes “CAR” and “ROAD”. We have noticed vibration effects with “under the bridge” camera and camera 73. Therefore, during the training process, we have added sixteen frame cars (six from “under the bridge” and 10 from camera 73) with vibrating effects into our training datasets. We had halation effects with camera 66, especially during night time. To address this issue, we added forty-seven halation effected training cars into our training datasets. 

### 4.2. Testing Data

**Figure 7:**
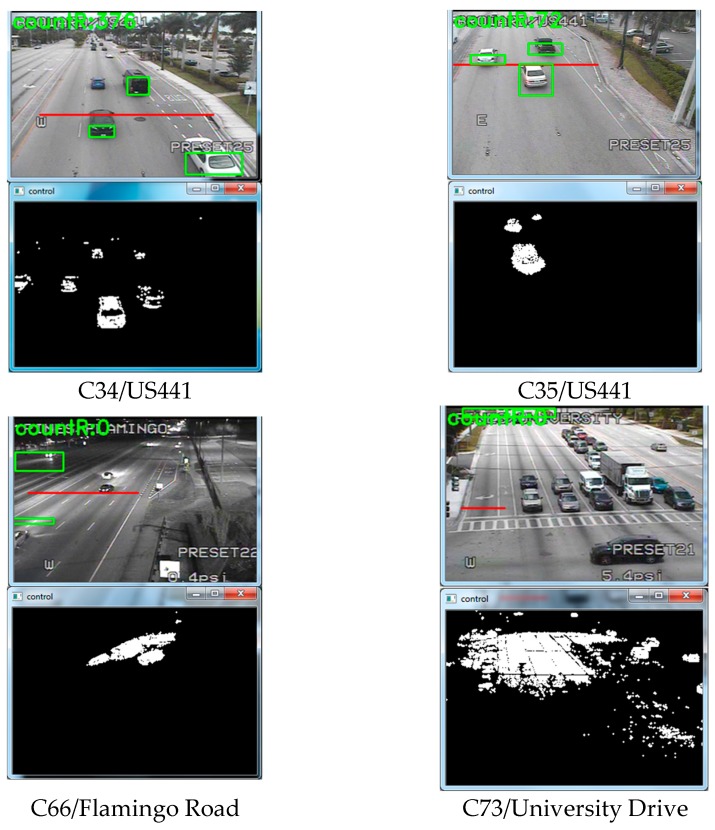

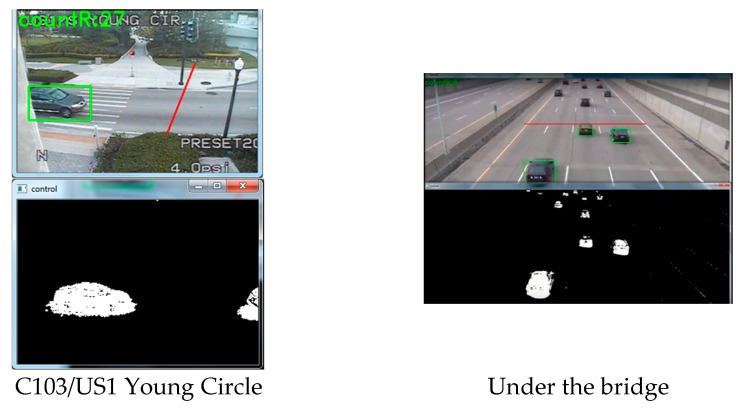
Background subtraction method results.

**Figure 8:**
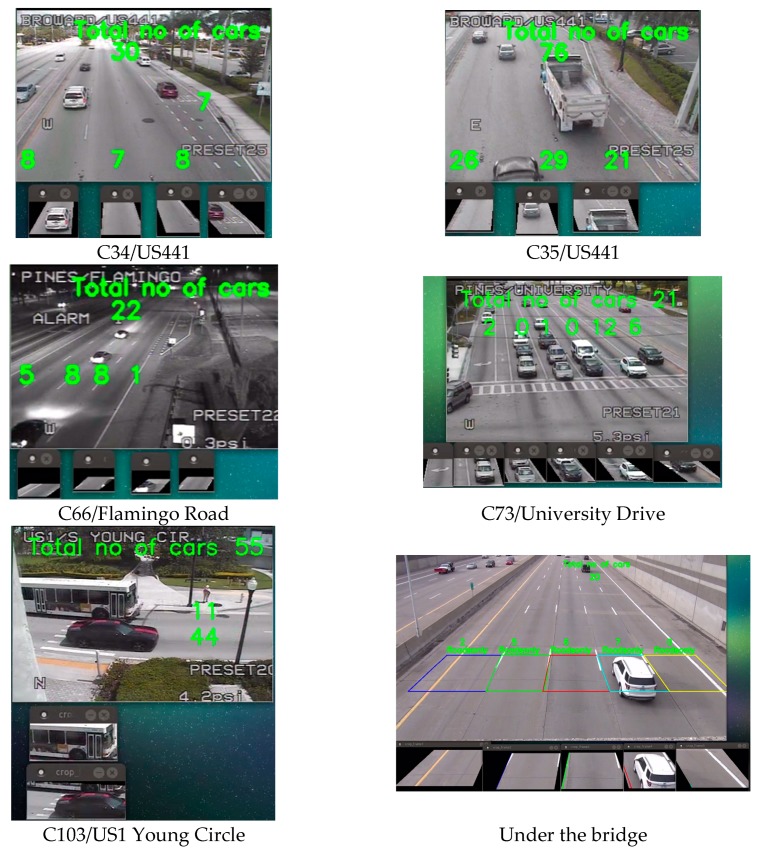
OverFeat network results.

We used six traffic videos for our study. Figure 7 and Figure 8 gives a glimpse of the videos. Camera 34 and 35 is placed at US441. It has three through lanes and one right lane. Camera 66 is placed at Flamingo Road, which contains four through lanes. We have used night time clip for camera 66. Camera 73 is placed at an intersection of University Drive, which contains three through lanes, one right turn and two left turns. This video was taken on a cloudy day and the weather was windy. This is why there was a shaking effect at the clip. Camera 103 is a side view camera that is placed at the US1 Young Circle. It contains two lanes. Finally, we had a high-resolution camera which is placed under a bridge [28], which contains five through lanes. All the aforementioned cameras have a downward inclination and the sides and top are cover with a large camera housing. This prevents any major complications with direct sun angles.

## 5. Result and Discussion

We tested the BSM and OverFeat framework on six different traffic cameras. The results are shown in Figure 7 for BSM and in Figure 8 for OverFeat framework. The footages were collected at separate times of the day. BSM works well when the weather condition and camera resolution is good. Table 2 shows the result set for the experiment where column one indicates the camera name, column 2 indicates the time footage has been taken, column 3 indicates the manual counts, column 4 indicates the result from a commercial software Placemeter, column 5 indicates the result from BSM and column 6 indicates the result from OverFeat framework. Cameras 34 and 35 have a similar kind of view. In addition, the results are consistent for both of the cameras. Placemeter and BSM’s performances are average for these two cameras, but OverFeat framework has shown excellent accuracy (>96%). The footage has been taken during the day and at night for camera 66. During the daytime, the accuracy was acceptable for BSM; however, during the nighttime, it fails completely. The reason behind the failure is the car lights. When there are multiple cars, the lights of all the cars are moving simultaneously, and it produces an effect that looks like a moving cloud of light. At that time, the BSM cannot identify the moving cars. However, the OverFeat framework achieves impressive 99.96% accuracy. Camera 73 has a very complicated view. This camera has right turn, left turn and go through also. The traffic stops sometime at the intersection because of the signal. The footage that we obtained for this camera has cloudy weather. Thus, counting with camera 73 was very challenging. From the Figure 7, it reveals that, for camera 73 when a cloud is moving, the background difference image contains moving shadows. Under the moving shadow, the algorithm can’t differentiate the moving cars. This is why experiment with camera 73 was very challenging. For this camera, Placemeter and BSM showed very average accuracy (57.77% and 52.92%, respectively), but OverFeat framework has shown 94.44% accuracy, which is impressive. Camera 103 is a side view camera. Because of that, a moving object stays longer at the field of view. An algorithm like BSM will tend to generate multiple centroids in this situation, which will confuse the algorithm to create multiple fake cars. In this situation, the OverFeat framework also showed an accuracy of 92.85%. We used a high-resolution video under a clear weather condition and labeled “under the bridge” to perform both the BSM algorithm and OverFeat framework. Both algorithms achieved 96.15% accuracy for this footage. The accuracy formula that we used is:

(5) $$\mathrm{Accuracy}\;=1-\left(\frac{\left|\mathrm{manual}\;\mathrm{counts}-{\mathrm{algorithm}}^{'}s\;\mathrm{counts}\right|}{\mathrm{manual}\;\mathrm{counts}}\right).$$

**Figure 9:**
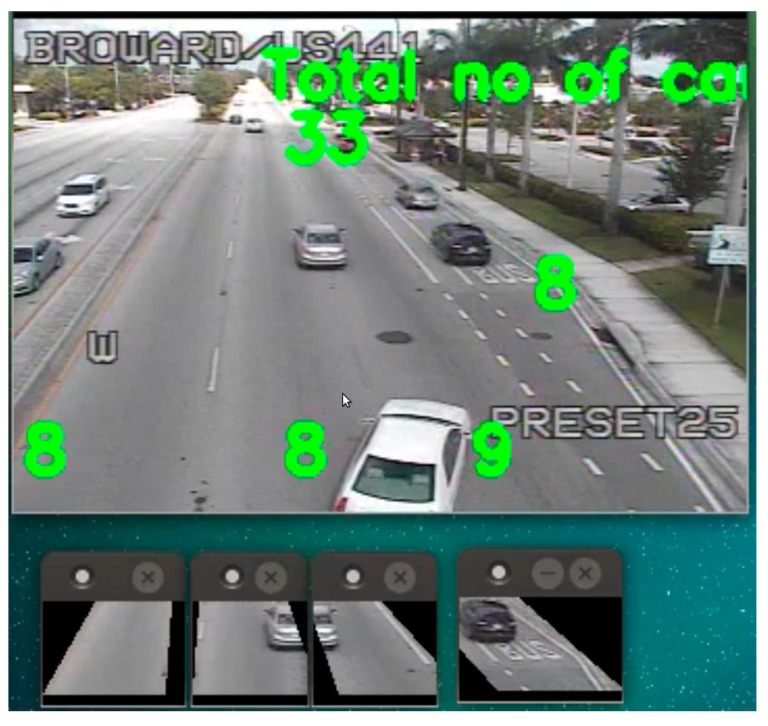
The double counting issue, when a car moves between two RIOs.

From Figure 9, we can see that when a car drives between the ROIs, both of the ROIs count the same car. Therefore, the same car was counted twice. We will continue to improve this framework in the near future. In addition, testing the car accounting system under rainy and heavy windy conditions could be another challenge to resolve in the future.

## 6. Conclusions

We developed and implemented two algorithms, BSM and OverFeat Framework, using the Python language for automatic car counting. The accuracy was evaluated by comparing with the manual counting. The comparative study was conducted between BSM, OverFeat framework, and Placemeter. For all of the videos, the OverFeat framework outperformed the other two methods (average accuracy for Placemeter is 67.69% and BSM is 63.14%, respectively) and reached an average accuracy of 96.55%. From the results, it showed that OverFeat has a great potential to be used in the field of traffic monitoring. The main challenge that we found during the experiments of the OverFeat Framework is how to define the ROIs. Defining ROI needs a large amount of manual effort. If the automatic determination of the ROIs is available, the OverFeat Framework will become an ideal solution for the car counting system. 

## Figures and Tables

**Table 1:** Architecture of the OverFeat network.

Layer	1	2	3	4	5	6	7	Output 8
Stage	conv + max	conv + max	conv	conv	conv + max	full	full	full
#channels	96	256	512	1024	1024	3072	4096	1000
Filter size	11 × 11	5 × 5	3 × 3	3 × 3	3 × 3	-	-	-
Conv. stride	4 × 4	1 × 1	1 × 1	1 × 1	1 × 1	-	-	-
Pooling size	2 × 2	2 × 2	-	-	2 × 2			
Pooling stride	2 × 2	2 × 2	-	-	2 × 2	-	-	-
Zero-Padding size	-	-	1 × 1 × 1 × 1	1 × 1 × 1 × 1	1 × 1 × 1 × 1	-	-	-
Spatial input size	231 × 231	24 × 24	12 × 12	12 × 12	12 × 12	6 × 6	1 × 1	1 × 1

**Table 2:** Accuracy assessment of the algorithms.

Camera	Time Duration (Local Time)	Manual Counts	Placemeter	BSM	OverFeat
C34	10:00–11:00	879	582 (66.21%)	597 (67.91%)	910 (96.47%)
	18:00–19:00	2075	1467 (70.96%)	1335 (64.33%)	2120 (99.97%)
C35	07:00–08:00	1862	1332 (71.53%)	2236 (79.91%)	1902 (97.85%)
C66	11:00–12:00	1978	1393 (70.42%)	1674 (84.63%)	1942 (98.17%)
	23:00–00:00	549	335 (61.02%)	108 (19.67%)	566 (99.96%)
C73	11:00–11:10 (for 10 min)	270	156 (57.77%)	151 (52.92%)	255 (94.44%)
C103	07:00–08:00	210	145 (69.04%)	372 (22.85%)	225 (92.85%)
	11:00–12:00	579	432 (74.61%)	463 (79.96%)	619 (93.09%)
Under the bridge	09:00-09:01 (1 min)	52	-	50 (96.15%)	54 (96.15%)
Average	-	-	67.69%	63.14%	96.55%
